# Effects of *Allium cepa* and Its Constituents on Respiratory and Allergic Disorders: A Comprehensive Review of Experimental and Clinical Evidence

**DOI:** 10.1155/2021/5554259

**Published:** 2021-09-13

**Authors:** Sima Beigoli, Sepideh Behrouz, Arghavan Memarzia, Seyyedeh Zahra Ghasemi, Marzie Boskabady, Narges Marefati, Farzaneh Kianian, Mohammad Reza Khazdair, Hesham El-Seedi, Mohammad Hosein Boskabady

**Affiliations:** ^1^Endoscopic and Minimally Invasive Surgery Research Center, Mashhad University of Medical Sciences, Mashhad, Iran; ^2^Applied Biomedical Research Center, Mashhad University of Medical Sciences, Mashhad, Iran; ^3^Department of Physiology, Faculty of Medicine, Mashhad University of Medical Sciences, Mashhad, Iran; ^4^Dental Materials Research Center and Department of Pediatric Dentistry, School of Dentistry, Mashhad University of Medical Sciences, Mashhad, Iran; ^5^Department of Pediatric Dentistry, School of Dentistry, Mashhad University of Medical Sciences, Mashhad, Iran; ^6^Department of Physiology, School of Medicine, Tehran University of Medical Sciences, Tehran, Iran; ^7^Cardiovascular Diseases Research Center, Birjand University of Medical Sciences, Birjand, Iran; ^8^Department of Medicinal Chemistry, Uppsala University, Biomedical Center, Box 574, SE-751 23, Uppsala, Sweden; ^9^International Research Center for Food Nutrition and Safety, Jiangsu University, Zhenjiang 212013, China; ^10^Al-Rayan Research and Innovation Center, Al-Rayan Colleges, Medina 42541, Saudi Arabia

## Abstract

The health benefits of *Allium cepa* (*A. cepa*) have been proclaimed for centuries. Various pharmacological and therapeutic effects on respiratory, allergic, and immunologic disorders are shown by *A. cepa* and its constituents. Flavonoids such as quercetin and kaempferol, alk(en)yl cysteine sulfoxides including S-methyl cysteine sulfoxide and S-propyl cysteine sulfoxide, cycloalliin, thiosulfinates, and sulfides are the main compounds of the plant. *A. cepa* displays broad-spectrum pharmacological activities including antioxidant, anti-inflammatory, antihypertensive, and antidiabetic effects. Our objective in this review is to present the effects of *A. cepa* and its constituents on respiratory, allergic, and immunologic disorders. Different online databases were searched to find articles related to the effect of *A. cepa* extracts and its constituents on respiratory, allergic, and immunologic disorders until the end of December 2020 using keywords such as onion, *A. cepa*, constituents of *A. cepa*, therapeutic effects and pharmacological effects, and respiratory, allergic, and immunologic disorders. Extracts and constituents of *A. cepa* showed tracheal smooth muscle relaxant effects, indicating possible bronchodilator activities or relieving effects on obstructive respiratory diseases. In experimental animal models of different respiratory diseases, the preventive effect of various extracts and constituents of *A. cepa* was induced by their antioxidant, immunomodulatory, and anti-inflammatory effects. The preventive effects of the plant and its components on lung disorders induced by exposure to noxious agents as well as lung cancer, lung infection, and allergic and immunologic disorders were also indicated in the experimental and clinical studies. Therefore, this review may be considered a scientific basis for development of therapies using this plant, to improve respiratory, allergic, and immunologic disorders.

## 1. Introduction

*Allium cepa* L. *(A. cepa)* or onion species are used as vegetables and employed in traditional medicine as therapeutic agents [1–3]. Onion is a perennial plant that is cultivated in almost all countries, mainly in moderate climate regions such as Iran [4–6]. There are various onion varieties including white, yellow, purple, red, and green onions, which vary in color, and sweet and nonsweet onions differing in taste [4, 7, 8]. The stems of the plant are green, its leaves are hollow, its height can reach 1 m, and it has small white or purple flowers. The bulb of the plant which grows under the ground is used for medical or food purposes and as a spice with an exquisite odor and taste [9].

Onion bulbs have been used as a food, spice, and herbal remedy since ancient times by people around the world, and several therapeutic properties were described for this plant [6, 10].

*A. cepa* has been considered a famous herbal medicine in Ayurveda for several indications such as fever, dropsy, catarrh, and chronic bronchitis, in the forms of decoction, infusion, fresh juice, and raw, cooked, or roasted bulb [11]. The use of *A. cepa* species in the treatment of angina pectoris, dyspnea, dysentery, cough, and bronchial obstruction has been noted in Chinese pharmacopoeia [11]. In the ancient times, onion was used for various healing purposes in Egypt [12]. Furthermore, *A. cepa* tea has been used for treatment of fever, headache, cholera, dysentery, common cold, and arthritis in Chinese medicine [13]. The effect of *A. cepa* on respiratory diseases was also indicated in ancient Iranian traditional medical books [14–16].

Asthma is an inflammatory disease of the lungs that makes breathing difficult and limits physical activities. Various cells such as T cells, mast cells, basophils, macrophages, and eosinophils are involved in the inflammatory processes of asthma. Among these cells, higher numbers of eosinophils are a characteristic feature of asthma. Total white blood cell (WBC) and eosinophil counts were enhanced in sensitized animals and asthmatic patients. Therefore, attenuation of the inflammation is essential for the treatment of asthma [17].

Chronic obstructive pulmonary disease (COPD) is a major cause of morbidity and mortality worldwide that results in substantial social and economic burdens. COPD is a heterogeneous disease with both extrapulmonary and pulmonary components. Obstructive lung diseases are often diagnosed based on symptoms and decreased pulmonary function tests (PFT). Obstructive lung diseases are managed by avoiding triggers such as dust and smoking, use of bronchodilators to control symptoms, and suppression of lung inflammation [18].

Lung cancers are also among lung disorders which, despite advances in our understanding of risk factors involved, its development, and its immunologic control and treatment options, remains a leading cause of death. Tobacco smoking is the predominant risk factor for lung cancer development. The known risk factors for lung cancer include behavioral, environmental, and genetic risk factors, all of which play a part in tumor development. The low overall 5-year survival rate for lung cancer patients has only minimally changed in decades [19].

Acute respiratory infections account for 20–40% of outpatient and 12–35% of inpatient attendance in a general hospital. Upper respiratory tract infections including nasopharyngitis, pharyngitis, tonsillitis, and otitis media constitute 87.5% of the total episodes of respiratory infections. The vast majority of acute upper respiratory tract infections are caused by viruses. Common cold is mainly caused by viruses and does not require antimicrobial treatment unless it is complicated by acute otitis media with effusion, tonsillitis, sinusitis, and lower respiratory tract infection. Sinusitis is commonly associated with common cold. Most instances of rhinosinusitis are viral, and therefore, they resolve spontaneously without antimicrobial therapy. The most common bacterial agents causing sinusitis are *S. pneumoniae, H. influenzae, M. catarrhalis, S. aureus*, and *S. pyogenes* [20]. Worldwide, tuberculosis is an important cause of pneumonia. Other pathogens such as viruses and fungi can cause pneumonia and severe acute respiratory syndrome and pneumocystis pneumonia. Pneumonia may develop complications such as lung abscess, a round cavity in the lung caused by the infection, or may spread to the pleural cavity [21].

Allergic conditions/disorders have increased during the last three decades all over the world due to changes in environmental factors including increased allergens, air pollution, and infection diseases [22]. Changes in foods and their amount in the diet may also contribute to increased risk of respiratory and allergic diseases. In addition, the interaction of environmental and genetic factors can affect the immune system and lead to the development of allergic diseases [23]. Serious allergic disorders include respiratory and skin allergies in which the immune system reacts to familiar allergens and reexposure to these agents leads to a massive secretion of allergy-related mediators which cause allergic symptoms [24].

Drugs that are currently used for the treatment of respiratory disorders may cause adverse effects and lack a high therapeutic efficacy; thus, new drugs should be developed for the treatment of these diseases [25]. Two types of drugs used for the treatment of inflammatory and obstructive respiratory diseases are relieving drugs that reduce airway obstruction and preventive drugs that suppress lung inflammation [26, 27]. Several adverse side effects were reported for drugs typically used in the treatment of asthma and allergic rhinitis such as antihistamines, decongestants, anticholinergic, and corticosteroids, including sedation, impaired learning and memory, and cardiac arrhythmias [25]. Therefore, therapeutic strategies should seek to decrease the side effects of the currently prescribed drugs. In fact, several safe natural therapies such as *Urtica dioica*, bromelain, quercetin (Qt), N-acetyl cysteine, and vitamin C have been introduced for treatment of the abovementioned disorders [28]. The antiallergic effect of polyphenols found in foods and plants on different disease models and clinical trials are shown; polyphenols have shown anti-inflammatory, antioxidant, and immunomodulatory effects and could modulate allergic sensitization by interaction with proteins and inhibit mediator release [29]. Several studies also showed the preventive effect of derivatives from *A. cepa* such as Qt on respiratory disorders [30–32].

Treatments used against respiratory, allergic, and immunologic disorders with synthetic drugs do not fully cure these diseases and may cause various adverse side effects [33]. Therefore, using natural products such as some medicinal herbs, flavonoids, lactones, alkaloids, polysaccharides, diterpenoids, and glucosides, with immune-modulating and anti-inflammatory properties, may potentially help in treatment of respiratory, allergic, and immunologic disorders [34]. In fact, the effects of polyphenols on respiratory and allergic disorders such as atopic eczema, food allergy, and asthma were demonstrated [35]. Therefore, the effects of *A. cepa* and its constituents in respiratory, allergic, and immunologic disorders were reviewed in this article.

## 2. Constituents of *A. cepa*

*A. cepa* contains vitamins and minerals, sulfur amino acids, and a variety of secondary metabolites such as flavonoids (particularly flavonols and anthocyanin), phytosterols, and saponins [10]. Also, it is a rich source of phenolic acids, sulfur compounds (allicin), and various types of biological phytomolecules such as phenolic acids, thiosulfinates, anthocyanins, kaempferol, and glycosides [36, 37].

Onions contain two subgroups of flavonoids: (1) the anthocyanins that are responsible for red or purple color of some varieties and (2) flavanols such as Qt and its derivatives, which are responsible for the yellow varieties and brown color of the skin of onion. Another chemical group found in onion is the alk(en)yl cysteine sulfoxides (ACSOs), known as flavor precursors. The distinctive smell and taste of onions are due to the breakdown of ACSOs by the enzyme alliinase. Fructooligosaccharides are other types of phytochemicals in onions that mainly include inulin, kestose, nystose, and fructofuranosyl nystose [38].

**Figure 1:**
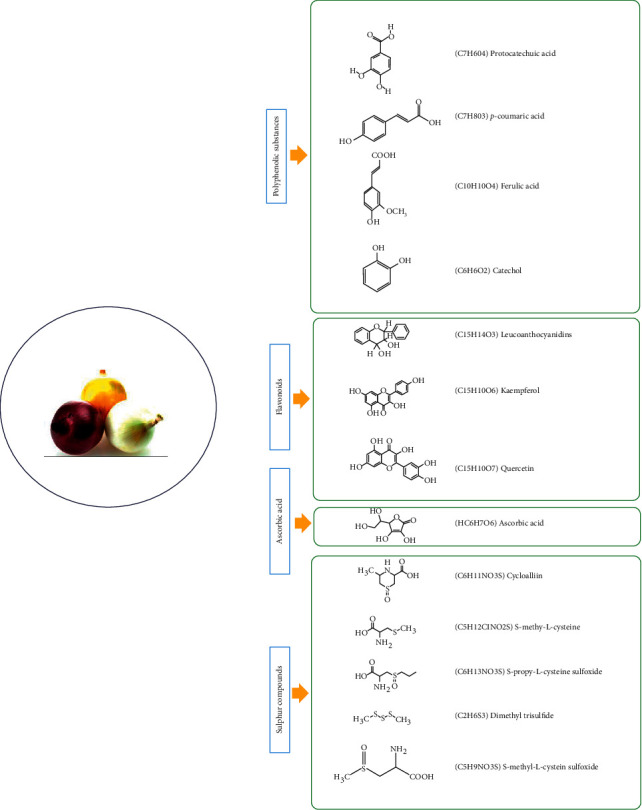
Chemical structure of the main constituents of *A. cepa*.

In general, constituents of onions are classified as follows:

  Polyphenolic substances: phenolic compounds in onions include protocatechuic, p-coumaric, ferulic acids, and catechol [39]. Onion phenolic acids are derived from benzoic acid or cinnamic acid. These phenolic acids help to create bitterness and aroma in the plant products [40].
  Flavonoids*:* onion contains the basic flavonoids groups such as catechins (flavan-3-ols), leucoanthocyanidins (flavan-3,4-diols), flavanones, flavanonols, flavonols, and anthocyanidins. The predominant flavonol in onions is Qt which is present in free and bound forms and together with glycosides shows an antioxidant activity [41]. Other flavonoids in onions include luteolin and kaempferol [42]. The highest amount of flavonols is found in red onion, for red anthocyanins in the form of glycosides cyanidin, peonidine, and pelargonidine.
  Ascorbic acid*:* ascorbic acid (vitamin C) is found in various amounts in a variety of vegetables and fruits. This water-soluble vitamin is reversible for the entire redox system [43, 44]. Vitamin C, Qt, and other active components of onions called isothiocyanates have anti-inflammatory effects [45].
  Sulfur compounds: there are many organic compounds in onions, including sulfur, which is responsible for the unpleasant onion odors. The main ingredient in onion flavor is propylene-L-cysteine sulfoxide, which is annoying to some animals. Other sulfur compounds in onions include *γ*-glutamyl peptides, S-substituted cysteines, and cycloaline, which are nonvolatile and have no effect on onion taste [46]. Onion components and their biological activities are shown in Table 1. The chemical structures of the main constituents of the plant are presented in Figure 1.

## 3. Methods

Literature review was carried out by searching the databases PubMed, Scopus, and Web of Science using the following key terms: “*Allium cepa*,” “onion,” “flavonoid,” “quercetin,” “phenolic compounds,” “therapeutic effects,” “pharmacological effects,” “allergic disorders,” and “respiratory disorders” from 1984 to the end of 2020. Articles about the effects of *A. cepa* on respiratory and immunologic disorders, lung cancer, and lung infection written in the English language from 1984 to the end of 2020 have been incorporated in this article. The reference lists of the collected articles were also investigated to recognize further studies.

## 4. Traditional and Pharmacological Effects

Various pharmacological effects such as antidiabetic, antihyperglycemic, antiparasitic, antifungal, antimicrobial, antiplatelet, anti-inflammatory, antioxidant, and antispasmodic properties were reported for the extracts of *A. cepa* and its different constituents [28, 31, 42, 47–52]. The preventive effects of the extracts of *A. cepa* on the vascular and heart diseases [53], neurodegenerative and antidepressant disorders [8], and cataract formation as well as improving effects on kidney function were also reported [6, 54]. *A. cepa* has carminative and expectorant effects and could improve dysmenorrhea, vertigo, fainting, migraine, wounds, scars, keloids, pain and swelling after bee sting, bruises, earache, jaundice, and pimples [29]. *A. cepa* also showed antitumor activity [29] and could decrease the risk of stomach carcinoma [55] and inhibit proliferation of leukemia HL60 cells [56, 57].

The effect of *A. cepa* and its derivatives on respiratory diseases includes a relaxant effect on the tracheal smooth muscle (TSM) [58–61], a modulatory effect on the immune system [61], tracheal responsiveness and lung inflammation [17] in sensitized rats, antiasthmatic effects on a murine model of asthma [30], and antiasthmatic properties [62, 63]. The World Health Organization (WHO) also recommended using the *A. cepa* extract for the treatment of diseases including common colds, coughs, asthma, bronchitis, and allergic disease [64]. Onion animal extract showed antiasthmatic effect through leukotriene or thromboxane biosynthesis and histamine release inhibition [31].

The antiallergic potential of the extracts of *A. cepa* [31] and its flavonoid quercetin was reported in previous studies [32, 65]. It was also shown that the antiallergic potential of quercetin is similar to Chinese herbal formula (Food Allergy Herbal Formula) which inhibits anaphylaxis to peanuts in mice [66]. The anti-inflammatory and antiallergic properties of quercetin on respiratory and food allergies were also shown [67, 68]. Antiallergic [69], neuroprotective [70], anti-inflammatory, and antioxidant activities [71] were shown for derivatives of *A. cepa* including flavonoids, organosulfur, and phenols.

The polyphenol compounds present in onions showed stimulating effects on the immune system in the aging process [72, 73], and some phenolics in onions showed antiplatelet properties [74]. The antimicrobial effects of protocatechuic, p-coumaric, ferulic acids, catechol [7], and kaempferol [39] were also reported. Kaempferol also showed detoxifying, apoptotic, antineoplastic [75], anti-inflammatory, and antioxidant activities [76, 77].

The sulfur compounds possess antibacterial, antifungal, antitumor, and antilarval effects [78]. Therefore, onion sulfur compounds can be considered natural preservatives to control microbial growth [79]. Luteolin, Qt, and baicalein could inhibit the secretion of granulocyte macrophage colony-stimulating factor in human cultured mast cells, suppress the secretion of leukotrienes, prostaglandins D2, and histamine, and inhibit tumor necrosis factor- (TNF-) *α* and IL-6 in bone marrow-derived culture fluid cells [80]. Due to antioxidant ability and cholesterol level-controlling properties of flavonoids and Qt present in onion, this plant is used in prevention and treatment of cardiovascular diseases [81, 82]. The protective effect of Qt on oxidative stress in Alzheimer's disease and neurodegenerative disorders was also demonstrated [83]. In addition, onion flavonoids could suppress proinflammatory factors of hematoma and improve the symptoms of intracerebral hemorrhage by inhibiting the activation of microglia [84]. Therefore, the onion extract has proven antiallergic and anti-inflammatory effects mediated via diverse mechanisms.

## 5. Bronchodilatory Effect of *A. cepa* and Its Constituents and Relieving Effects of These Agents on Obstructive Pulmonary Disorders

### 5.1. Effects of the Plant Extracts and Essential Oil

#### 5.1.1. Experimental Evidence

In a study, *A. cepa* extracts (AcE), (2, 4, 8, 16, 32, and 64 mg/ml) showed concentration-dependent relaxant effects on tracheal smooth muscle (TMS) of rats contracted by KCl or methacholine. There was no significant difference in the relaxant effects of AcE between nonincubated and incubated tissues with glibenclamide, atropine, chlorpheniramine, and indomethacin. EC50 values of AcE in TSM incubated with propranolol and diltiazem were significantly lower than nonincubated tissues. The relaxant effects of different concentrations of the AcE were not significantly different from those of theophylline. The concentrations of AcE and theophylline were significantly correlated with their relaxant effects. In TSM incubated with propranolol and diltiazem, concentration ratio minus one (CR-1) values were positive. The results showed a potent relaxant effect of the plant on TSM which was possibly induced by *β*2-adrenergic stimulation and/or calcium channel blockade. These findings suggest a possible bronchodilatory effect for AcE in obstructive pulmonary diseases [61].

Mandukhail et al. reported dose-dependent (3–30 mg/kg) reduction of carbachol (CCh), (1 mg/kg)-induced bronchoconstriction by a flavonoid-rich hydroacetone AcE peel, similar to the effect of aminophylline in rats. In guinea pigs also, the AcE (0.3–3 mg/mL) relaxed both CCh (1 *μ*M) and high K^+^-induced contraction of TSM concentration dependently and shifted the isoprenaline-induced relaxation concentration-response curves to the left, similar to effect of papaverine. The results indicated that the relaxant effect of the AcE on TSM is mediated through inhibition of Ca^2+^ channels and phosphodiesterase enzyme-like mechanism, suggesting red onion peel as a bronchodilatory agent in obstructive pulmonary diseases [85].

Benzyl-isothiocyanates (BITC, 15, 30, 75, and 150 mg/kg), a component of onion, also decreased bronchial obstruction dose dependently. In addition, ethyl-isothiocyanates and allyl-isothiocyanate similarly inhibited bronchial obstruction. However, no antagonistic effects of ethanolic AcE 10 *µ*l/ml given orally to the animals 30 min prior to allergen inhalation challenges on histamine- or acetylcholine (ACh-) induced bronchial obstruction were shown [86].

### 5.2. Effects of the Plant's Constituents

#### 5.2.1. Experimental Evidence

The relaxant activities of various concentrations of Qt (3.5, 7.5, and 15 *μ*g/ml) on TMS of A/J mice precontracted with CCh were reported [48]. It was shown that the ethyl acetate fraction of Qt (10 *μ*M–1.0 mM) prevents Ca^2+^-permeant L-type voltage-dependent Ca^2+^ channels (LVDCCs), short transient receptor potential channel 3 (TRPC3), and stromal interaction molecule (STIM)/Orai channels, leading to inhibition of precontraction of TSM in mice. In addition, ACh-induced contraction of TSM was inhibited by Qt. Therefore, Qt is able to inhibit Ca2+-permeant LVDCCs, TRPC3, and STIM/Orai channels that relax the precontracted TSM. These results suggest that Qt could be used to develop a new bronchodilator drug to treat obstructive lung disorders such as asthma and COPD [87].

In an *in vitro* study, Qt (100 nM-1 mM) acutely and concentration dependently relaxed TSM precontracted with ACh. Qt (50 *μ*M) also markedly potentiated isoproterenol-induced relaxations of TSM. Qt directly mitigated phospholipase C activity, inositol phosphate synthesis, and intracellular calcium responses to Gq-coupled agonists. In an *in vivo* study, nebulized Qt (100 *μ*M) also considerably attenuated methacholine-induced airway resistance. These results indicated that the bronchodilatory effects of QT were possibly mediated by selective inhibition of phosphodiesterases-4 (PDE_4_), suppression of degradation of cyclic adenosine monophosphate, and increase in PKA signaling in TSM or through *β*-receptor stimulation [88].

The effects of Qt on ovalbumin- (OVA-) sensitized conscious guinea pigs and airway obstruction induced by histamine and ACh were examined using whole body plethysmography; results showed significant bronchodilation induced by Qt at 20 mg/kg. These results suggest the possible use of Qt for the treatment of airways obstruction because of its bronchodilatory effects *in vivo* and *in vitro* [89].

In a similar study, the relaxant effects of Qt on both CCh and electrical field stimulation- (EFS-) induced TSM precontraction were observed. The results also showed more prominent relaxant effects for Qt in TSM contracted by EFS than that contracted by CCh, suggesting a presynaptic effect for Qt in addition to the postsynaptic effect, as revealed by the inhibitory action of Qt on CCh-induced contractions. The inhibitory effect of Qt on contractions induced by EFS was not affected by phentolamine plus propranolol, tachykinin NK_1_ and NK_2_ receptor antagonists, and capsaicin treatment or by the proteolytic enzyme *α*-chymotrypsin. In contrast, the nitric oxide synthase inhibitor *N*^G^-nitro-L-arginine methyl ester significantly decreased the inhibitory effect of Qt on contractions induced by EFS [59].

Concentration-dependent relaxant effects of Qt were shown on ACh or histamine-contracted human airways smooth muscle (HASM). In addition, K^+^ and Ca^2+^ concentration-contraction curves were inhibited by incubation of HASM with increasing concentrations of Qt. Qt also enhanced the relaxant effects of isoprenaline or sodium nitroprusside concentration dependently. These findings indicated that the bronchodilatory effects of Qt are possibly mediated through increasing cyclic nucleotide levels and altering availability of Ca^2+^ to the contractile machinery [90].

In the sensitized guinea pigs to OVA, Qt 20 mg/kg administered 30 minutes before the contractile agents significantly inhibited airway contraction induced by cumulative doses of histamine or ACh, indicating the bronchodilatory effect of Qt on allergic asthma [60].

**Figure 2:**
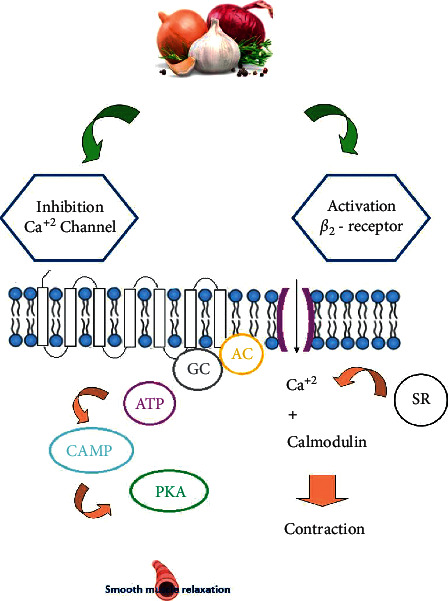
The possible mechanisms of the relaxant effect of *A. cepa* and its constituents on the tracheal smooth muscle.

Overall, the experimental studies showed the relaxant effect of AcE and its constituent, Qt, on precontracted TSM induced by various smooth muscle contractile agents. The possible mechanisms of the relaxant effect of AcE or Qt on TSM are *β*2-adrenoceptors stimulation and/or inhibition of muscarinic and histamine H_1_ receptors, calcium channel blocking, and phosphodiesterase enzyme mechanisms. These results suggest the possible bronchodilatory effects of the plant and its constituent on obstructive respiratory diseases but further clinical studies are needed to examine this effect in asthma, COPD, or other obstructive pulmonary disorders. The possible bronchodilatory effects of *A. cepa* and its constituents are shown in Table 2, and mechanisms shown to underlie these effects are presented in Figure 2.

## 6. Preventive Effects of *A. cepa* and Its Constituents on Asthma

### 6.1. Effects of the Plant Extracts and Essential Oil

#### 6.1.1. Experimental Evidence

The AcE (35, 70, and 140 mg/kg b.w.) and dexamethasone (1.25 *μ*g/mL) effects on oxidants, antioxidants, and immunological markers in the bronchoalveolar lavage fluids (BALF) of OVA-sensitized rats revealed concentration-dependent improvement of these markers in treated groups. In addition, the effect of *A. cepa* extract was similar to the effect of dexamethasone [10].

In a similar study, the adjuvant effects of AcE (150 and 300 mg/kg b.w.) and dexamethasone (1 mg/kg) on OVA-sensitized Wistar rats were examined. Eosinophil and lymphocyte in the blood and the BALF of the asthmatic group were significantly increased but decreased in the AcE-treated groups, indicating the reduction of cellular infiltration and lung inflammation of AcE-treated asthmatic rats [91].

Ghorani et al. also demonstrated that aqueous-alcoholic AcE (0.175, 0.35, and 0.7 mg/mL) and dexamethasone (1.25 *μ*g/mL) administration to OVA-induced asthmatic rats during the sensitization period reduced tracheal responsiveness, lung inflammatory cells, and phospholipase A2 (PLA2) level in the BALF of the animals [17].

### 6.2. Effects of the Plant's Constituents

#### 6.2.1. Experimental Evidence

Antiasthmatic effects of the constituents of *A. cepa* were shown to be mediated through reduction of oxidative markers such as malondialdehyde (MDA), inflammatory mediators such as nuclear factor kappa B (NF-*κ*B), prostaglandin D_2_ (PGD_2_), leukotrienes, and granulocyte macrophage-colony stimulating factor (GM-CSF), elevation in antioxidants such as superoxide dismutase (SOD), and suppression of T helper (Th) 2-type synthesis of cytokines such as IL-4 and IL-13 [22, 92].

In asthmatic mice, Qt treatment markedly reduced airway hyperresponsiveness and inflammatory cell numbers in the BALF, inhibited matrix metalloproteinase (MMP) 9 and GATA-3 mRNA levels in the lung tissues, and improved Th1/Th2 balance (decreased Th2 cytokines IL-4 and IL-5 but increased Th1 cytokine interferon gamma (IFN-*γ*)) [32].

The AcE and the constituents of onion, mainly Qt, decreased total and differential WBC in the blood and BALF of animals sensitized with OVA (an animal model of asthma). Oxidant markers such as MDA was reduced, but antioxidants including CAT and SOD were increased in asthmatic animals due to treatment with AcE and Qt. Serum and BALF levels of PLA2, NF-*κ*B, PGD_2_ leukotrienes, and GM-CSF were also decreased due to treatment with AcE and Qt. Treatment with the plant and its constituents also decreased tracheal responsiveness and lung pathological changes in the sensitized animals. Th2-type cytokine (such as IL-4 and IL-13) synthesis was decreased, but Th1 cytokine IFN-*γ* was increased and Th1/Th2 balance was improved in asthmatic animals treated with AcE and the constituents of the plant [93].

Other active components of onions including thiosulfinates and sulfines (sulfinyl disulfides) are able to activate cyclooxygenase and 5-lipoxygenase pathways which initiate eicosanoid metabolism. Thus, these constituents might be responsible for anti-inflammatory and antiasthmatic properties of the onion extracts [94].

Treatments with kaempferol attenuated the Th2-driven allergic airway disease in an experimental model of asthma by decreasing production of IL-5 and IL-13 and amelioration of airway hyperresponsiveness (AHR) induced by OVA challenge. Kaempferol also inhibited IgE-mediated release of proinflammatory mediators from human mast cells [95]. The preventive effect of Qt (3.5, 7.5, and 15 *μ*g/ml) on cytokine levels in spleen cell culture supernatants showed a reduction in the production of inflammatory cytokines in *Blomia tropicalis*- (BT-) sensitized A/J mice. Treatment with Qt (30 mg/kg) reduced the total number of cells in the BALF and erythropoietin (EPO) in the lung. These results demonstrate a reduction in the production of inflammatory cytokines and total number of cells in the BALF and EPO in the lungs by treatment with AcE or Qt [30].

#### 6.2.2. Clinical Evidence

The results of several epidemiological studies suggest that consumption of Qt is beneficial for asthma therapy. Moreover, clinical trials on Qt have shown its ameliorative effects on symptoms related to asthma. Protective effects of Qt consumption on asthma incidence have been demonstrated by epidemiological and population-based case-control studies [36, 37, 96].

It was reported that diphenylthiosulfinate, a constituent of onion, inhibits the chemotaxis of human granulocytes induced by formyl-methionine-leucine-phenylalanine in a dose-dependent manner (0.1–100 mM) *in vitro*. The highest activity found for this agent was higher than that of prednisolone. Therefore, the anti-inflammatory properties of the onion extracts are related, at least in part, to its constituent, thiosulfinates, and this agent could be a candidate for the treatment of bronchial asthma [97].

Therefore, these results showed that *A*. *cepa* and its constituents could be considered possible preventive treatments for asthma. The ameliorative effect of Qt on asthma symptoms and its protective effect on asthma incidence were shown in epidemiological and population-based case-control studies. The preventive effects of *A*. *cepa* and its constituents on asthma are shown in Table 3.

## 7. Effect of *A. cepa* and Its Constituents on Lung Cancer

### 7.1. Effects of the Plant Extracts and Essential Oil

#### 7.1.1. Experimental Evidence

Treatment with AcE (10 g/L) showed antiproliferative capacity, and there was an association between the concentration of the extracts and reduction of mitotic indices. Furthermore, the extract did not indicate antimutagenic and genotoxic activity. These effects might be related to the phenolic compounds found in the extracts of onion [98].

### 7.2. Effects of the Plant's Constituents

#### 7.2.1. Experimental Evidence

Treatment of human lung cancer cell line NCI-H209 with Qt glucuronides decreased cell viability, dose and time dependently, but increased cell cycle and the proportion of cells in G2/M phase and subG0/G1 phase. Qt glucuronides also increased the expressions of cyclin B, Cdc25c-ser-216-p, and Wee1 proteins, indicating G2/M arrest. Decrease of mitochondrial membrane potential, release of cytochrome c, upregulation of Bax, downregulation of Bcl-2, activation of caspase-3, and cleavage of poly(ADP-ribose) polymerase were seen following Qt treatment, demonstrating the induction of apoptosis [99].

Treatment of A549 cells with Qt reduced cell viability, DNA synthesis, and Bcl-2 level but increased Bax, Bad, and Bcl-x(L), dose dependently. Moreover, Qt induced cleavage of caspase-3, caspase-7, and poly ADP-ribose polymerase (PARP), inhibited Akt-1 and p-Akt-1, and phosphorylated the extracellular signal-regulated kinase (ERK) and MEK1/2 in a dose-dependent manner. These findings suggest that Qt is able to induce apoptosis in A549 lung carcinoma cells [100].

The effects of *A. cepa* and its constituents on lung cancer were shown in several studies. Nicotine is a main toxic component of cigarette smoke that contributes to the development of lung cancer in smokers. In this regard, the protective effect of *A. cepa* oil as an antioxidant in nicotine-administered rats was examined. Treatment with *A. cepa* oil (100 mg/kg b.w. for 21 days) increased catalase (CAT) and SOD activity in the lung tissue of rats exposed to nicotine [101]. Another study also demonstrated that exposure of animals to nicotine led to emphysematous air spaces, with thickened interalveolar septa, massive congestion, extravasation of red blood cells, inflammatory cellular infiltration, and fluid exudate that were all improved by AcE administration. MDA level also decreased, but antioxidant marker (SOD and CAT) levels were increased due to treatment with AcE in rats [33].

#### 7.2.2. Clinical Evidence

In nontumor lung tissue from 38 adenocarcinoma patients, Qt-rich food intake was negatively correlated with lung cancer risk which was not different between P450 or GST genotypes, gender, or histological subtypes and the correlation was stronger in smoker subjects (smoking >20 cigarettes a day). In addition, gene expression in the high Qt-rich food consumption group showed a higher upregulation of GSTM1, GSTM2, GSTT2, and GSTP1 but downregulation of specific P450 genes compared to the low consumption group. These data show an association between Qt intake, tobacco smoking, and lung cancer risk and a possible therapeutic effect of Qt on lung cancer [99].

Intake of a Qt-rich diet, in the tissue samples from 264 lung cancer cases (144 adenocarcinomas and 120 squamous cell carcinomas), differentiated miRNA expression profiles of the tumor suppressor let-7 family in adenocarcinomas. Carcinogenesis-related miR-146, miR-26, and miR-17 were also significantly differentiated due to Qt-rich diet. Among former and current smokers with adenocarcinoma, 33 miRs were also differentiated between highest and lowest Qt-rich food consumers. This study indicates the differential expression of biologically functional miRs in Qt-rich food consumers with adenocarcinoma and supports the therapeutic effect of Qt on lung cancer [102].

Overall, treatment with Qt affects different cancer cell lines through modulating cell viability and other molecular mechanisms indicating its therapeutic effect on lung cancer. Various clinical studies also support the effect of Qt on lung cancer. The effects of *A. cepa* and its constituents on lung cancer are summarized in Table 4.

## 8. Effects of *A. cepa* and Its Constituents on Lung Infections

### 8.1. Effects of the Plant Extracts and Essential Oil

#### 8.1.1. Experimental Evidence

The findings of Ziarlarimi et al.'s study showed that *Escherichia coli* (*E. coli*) was resistant to the aqueous extracts of AcE [103]. However, antibacterial activity of onion (50 mg/ml, twice daily for 7 days) has been shown, and it was indicated that the plant can be used in the treatment of bacterial diseases and as an immune booster to inhibit bacterial (*P. aeruginosa*) infections [104]. The decrement of gold nanoparticles synthesized with onion and inoculation of this combination affected *E. coli* in trypticase soy broth. Application of this combination to *E. coli* and incubation for a period of time caused cell lysis, showing antibacterial effect of the combination; thus, onion could be an effective candidate for sanitation of food and healthcare institutions [105].

The essential oil of *A. cepa*, at a concentration of 28.0 *μ*L/100 mL, showed a fungicidal effect on the growth of *Aspergillus carbonarius*, *Aspergillus wentii*, *Aspergillus versicolor*, *Penicillium brevicompactum*, *Penicillium glabrum*, *Penicillium chrysogenum*, and *Fusarium spp*. In addition, the plant exerted an inhibitory effect on *Aspergillus niger* and *Penicillium aurantiogriseum* [106].

#### 8.1.2. Clinical Evidence

In a clinical study, in viral flu patients with mild symptoms of cough, headache, and sputum production at the onset of disease, a simple home-based treatment (self-treatment) of an alternative approach with inhalation of a preparation of onion, garlic, or scallions improved all symptoms, suggesting application of these plants for treatment of mild virus-infected respiratory diseases at onset of the disease [107]. It was also indicated that *A. sativum* can combat COVID-19 infection by modulating immune system cells, reducing the production and secretion of proinflammatory cytokines, and affecting adipose tissue-derived hormone leptin with proinflammatory nature [108].

Treatment of *P. aeruginosa* with a high concentration of crude juices of garlic (*A. sativum*) and *A. cepa* showed low D-value, but the opposite was indicated for *S. aureus* [109].

### 8.2. Effects of the Plant's Constituents

#### 8.2.1. Experimental Evidence

In several studies, the effect of Qt on microbial, viral, and parasitological lung infections was shown. Supplementation of intranasal viral instillation with oral Qt significantly reduced superoxide radicals and lipid peroxidation levels, the number of infiltrating cells, and lung morphological changes [110].

Treatment of influenza virus- (A/Hong Kong/8/68) infected Swiss albino mice with Qt decreased the lipid peroxide levels and formazan-positive cells in these mice [111]. Qt-loaded poly D,L-lactide-co-glycolide (PLGA) nanoparticles (PQTs) showed antibacterial activity on *E. coli* and *Micrococcus tetragenus* mediated by disrupting bacterial cell wall integrity dose dependently; the effect was more prominent on *E. coli* than *M. tetragenus*. In addition, the antibacterial activity in mice was also shown with the absence of lung pathological changes in treated animals with PQTs [97].

Treatment of influenza virus- (A/Udorn/317/72(H3N2)) infected mice with Qt increased GSH, SOD, and pulmonary concentrations of CAT but did not affect the fall in vitamin E level in the infected mice. Therefore, Qt may be of therapeutic value in protecting the lung injury due to oxidative stress induced by influenza virus infection [106].

#### 8.2.2. Clinical Evidence

Treatment with vitamin C and Qt at doses of 30 or 40 mg/kg, BID, po, for 4 days was suggested for both prophylaxes in high-risk populations and for the treatment of COVID-19 patients as an adjunct to promising pharmacological agents such as convalescent plasma. In fact, Qt showed antiviral effects by interfering with virus entry and replication and protein assembly which were augmented by coadministration with vitamin C. Therefore, these two compounds could be promising candidates for both the prophylaxis and early treatment of virus respiratory tract infections, especially in COVID-19 [112]. It was also indicated that Qt inhibits various viral infection and replications at different stages without serious side effects and could be a promising drug for the treatment of the common cold [113].

The alleviating effects of antiviral, anti-inflammatory, and respiratory symptoms of Qt of nebulized 1 mL, Qt of 200 mg/mL, and 1 mL N-acetyl cysteine (NAC) (100 mg/mL, three times a day) were reported. Therefore, Qt formula could be recommended for further clinical study for COVID-19 and other viral pulmonary infections [114]. In patients with newly diagnosed destructive pulmonary tuberculosis, treatment with Qt and polyvinylpyrrolidone QP (5 g in 100 mL of 0.9% sodium chloride solution intravenously once a day for 10 days) resulted in quick reduction of the disease manifestation [115].

The reviewed papers indicated the effect of the extracts, essential oil, and the constituents of onion, mainly Qt, on viral, microbial, parasitic, and fungal infections in the lung. In experimental studies, the effect of onion on the lung infected with *E. coli* and *P. aeruginosa* was shown. The essential oil of *A. cepa* affected lung infections with various fungi including *Aspergillus carbonarius*, *Aspergillus wentii*, *Aspergillus versicolor*, *Penicillium brevicompactum*, *Penicillium glabrum*, *Penicillium chrysogenum*, *Fusarium spp*, *Aspergillus niger*, and *Penicillium aurantiogriseum*. Treatment with Qt improved influenza virus infection and its lung manifestation. Clinical studies showed beneficial effects of onion on symptoms of virus-infected flu including cough, headache, and sputum production. The effect of onion on the lung infected with *P. aeruginosa, S. aureus,* and *S. pneumonia* was also demonstrated. Treatment with Qt showed antiviral effects caused by interfering with virus entry and replication and protein assembly [116]. The effect of Qt on the treatment of COVID-19 patients was also suggested, and its effect on pulmonary tuberculosis was also demonstrated. The effects of *A. cepa* and its constituents on lung infections are summarized in Table 4.

## 9. Effects of *A. cepa* and Its Constituents on Allergic Disorders

The effects of *A. cepa* and its constituents on asthma were described in previous sections. The effect of the plant and its constituents on allergic and immunologic disorders is provided in this section.

### 9.1. Effects of the Plant Extracts and Essential Oil

#### 9.1.1. Experimental Evidence

In the Mediterranean diet, as well as in other diets, *A. cepa* is widely used in raw or cooked form [117, 118]. This plant is used for the treatment of allergic or upper airway diseases worldwide [17]. *A. cepa* is regarded as a folk remedy in almost all traditional and herbal medicines. Research studies also support the efficacy of the plant and showed positive effects of *A. cepa* and its constituents on immunologic and allergic disorders in animal studies [17, 119].

In allergic rhinitis in BALB/c mice induced by intraperitoneal administration of OVA and challenged with intranasal instillation of OVA, topical administration of *A. cepa* extract reduced allergic symptoms. The levels of OVA-specific IgE, IL-4, IL-5, IL-10, IL-13, and IFN-*γ* and eosinophil infiltration in nasal mucosa were significantly reduced due to treatment with onion extract. Hence, the topical administration of onion extract affects allergic symptoms through reducing Th1 and Th2 responses in allergic disorders [119].

In two other studies, *A. cepa* significantly inhibited IgE-induced histamine and beta-hexosaminidase release from RBL-2H3 cells [120]. In addition, the effects of onion peel hot water extract on cell viability, nitric oxide (NO), proinflammatory cytokines such as IL-6, TNF-*α*, and IL-1*β*, murine macrophage cell line, and RAW 264.7 from Balb/c mice with croton oil-induced mouse ear edema were examined. The level of NO, IL-6, TNF-*α*, and IL-1*β* production by OPHWE was decreased dose dependently compared with the lipopolysaccharide (LPS) group, indicating the anti-inflammatory and immunomodulatory activities of onion peel hot water extract. These results suggested that onion could be regarded as a candidate for the treatment of inflammatory and immune-dysregulatory disorders [121].

Dorsch et al. showed antiasthmatic effect of the *A. cepa* extract caused by improvement of leukotriene and thromboxane biosynthesis as well as histamine release. The efficacy of *A. cepa* in allergic diseases was indicated by improvement of leukotriene and thromboxane biosynthesis as well as histamine release [122, 123].The levels of TNF-*α* and IL-12 and phagocytosis in cultured peritoneal cells from mice were increased due to oral administration of the mucus of bunching onion. In addition, production of IFN-*γ* from spleen cells and natural killer (NK) activity were augmented in the treated groups, indicating increased natural immunity by oral onion.

The effect of a herbal fraction (ALC-02) from *A. cepa* on type I allergic reactions was shown to be mediated by inhibiting histamine release and reduction of intracellular calcium levels, as well as preventing systemic anaphylaxis and decreasing histamine levels and lipid peroxidation in compound 48/80-induced rat peritoneal mast cells. Carrageenan-induced rat paw edema, eosinophil peroxidase activity, and protein content in the BALF of OVA-sensitized mice ALC-02 were also reduced in the treated group. These findings showed the antiallergic property of *A. cepa* mediated by its potential antihistaminic, anti-inflammatory, antioxidant, and immunoregulatory activities [31].

In White Leghorn chickens, concentration-dependent inhibitory effects of garlic and onion extracts (0.8–409.6 *μ*g/ml) on cell proliferation and IL-2 and INF-*γ* gene expression of stimulated lymphocytes were shown which support the immunomodulatory effects of the two plants [124]. The effect of aqueous garlic and onion extracts (150 and 400 mg/kg/day, orally) during the last 8 weeks of fructose feeding (for 14 weeks) in thirty-day-old male Wistar rats was studied. Garlic and onion treatment decreased oxidative stress, increased eNOS activity, and reduced vascular cell adhesion molecule-1 (VCAM-1) expression which provided new evidence on anti-inflammatory and immunomodulatory effect of garlic and onion [125].

Atypical prostatic hyperplasia (APH) induced by subcutaneous (s.c.) injection of testosterone (0.5 mg/rat/day) and through smearing citral on the skin once every 3 days for 30 days was treated with onion suspension (75, 150, and 300 mg/kg/day; oral) and palmetto (100 mg/kg) was used as a positive control for 30 days. The results showed decreased IL-6, IL-8, and TNF-*α* which was dose dependent. These findings showed potential anti-inflammatory and immunomodulatory effects of the extract of onion as indicated by protective effects against APH induction in rats [48].

Application of *A. cepa* on the nasal cavity of BALB/c mice with allergic rhinitis revealed remarkable decreases in IgE and inflammatory cytokines IL-4, IL-5, IL-10, and IL-13. In addition, eosinophil infiltration into nasal turbinate mucosa was also considerably decreased [119]. In addition, it has been shown that *A. cepa* decreased vascular permeability leading to reduction of BALF protein exudation [31].

#### 9.1.2. Clinical Evidence

Several clinical studies demonstrated the effect of *A. cepa* on allergic and immunologic disorders. The effect of *A. cepa* supplementation (500 mg twice a day) on 419 cases with respiratory and allergic diseases showed a reduction in TNF-*α* and IL-6 [126].

The intranasal application of onion seed for 2 weeks in a cohort of 66 cases with allergic rhinitis reduced the nasal mucosal congestion, nasal itching, runny nose, sneezing attacks, turbinate hypertrophy, and mucosal pallor as well as IgE level and eosinophil count in nasal discharge during the first two weeks of treatment. Also, attenuation of the clinical symptoms of allergic rhinitis by stabilizing mast cell membranes was seen [127].

However, induction of allergic reaction to onion was also indicated in a number of studies. An episode of anaphylaxis following cooked onion ingestion was reported which was confirmed by skin test, and immuno CAP confirmed the IgE-dependent response to onion in this patient. In addition, only B cells were proliferated in response to onion extract. Therefore, cooked onion can induce severe allergic reactions, indicating the presence of thermostable components [2].

The effect of onion extracts on 2508 subjects with food intake-related symptoms and food hypersensitivity identified by the skin test, positive specific IgE, or provocation in 924 cases was examined. In 27 of these cases, food intake-related symptoms occurred following onion intake. Also, according to immunodetection results, an association between the symptoms and a specific lipid transfer protein (LTP) to the bulbs of onion was shown [128]. Therefore, allergic hypersensitivity to onions should not be underestimated and should be included in the diagnostic food allergy protocol [128].

### 9.2. Effects of the Plant's Constituents

#### 9.2.1. Experimental Evidence

Flavonoids in onions, such as Qt and kaempferol, showed various biological roles in health maintenance such as antiviral, antimicrobial, anti-inflammatory, anticancer, and immuno-modulatory activities [129, 130]. Various effects were reported for Qt such as stimulation of the immune system, antiviral activity, inhibition of histamine release, and suppression of proinflammatory cytokines and leukotrienes (e.g., IL-4). Qt also improved the Th1/Th2 balance, restrained antigen-specific IgE antibody formation, and suppressed lipoxygenase, eosinophil, and peroxidase activities and inflammatory mediator levels. Therefore, Qt with anti-inflammatory and immune-modulating properties could be regarded as a candidate in the treatment of asthma, allergic rhinitis, and restricted peanut-induced anaphylactic reactions [131].

A number of studies indicated that Qt treatment decreased LPS-induced IL-8 production in lung A54 cells and mRNA levels of TNF-*α* and IL-1*α* in glial cells, production of cyclooxygenase (COX), lipoxygenase (LOX), and Fc*ε*RI-mediated release of proinflammatory cytokines, tryptase, and histamine from human mast cells [132–134].

Qt could be a useful supplement for the management of eosinophil-mediated diseases, such as allergic rhinitis and asthma. Treatment with Qt (5.0, 7.5, 10.0, 15.0, 17.0, and 20.0 mg/kg, once a day for 3 weeks, orally) for *Mesocestoides corti* infection in BALB/c mice, suppressed eosinophil activation with a minimum concentration of 5.0 *μ*M but did not affect eosinophil growth or IgE hyperproduction [135].

Administration of isoquercitrin 15 mg/kg, Qt 10 mg/kg, or dexamethasone (1 mg/kg, s.c.) to BALB/c mice sensitized with OVA reduced eosinophil counts in the BALF, blood and lung parenchyma, neutrophil counts in the blood, and IL-5 levels in the lung homogenate (only in isoquercitrin-treated mice). In addition, Qt and isoquercitrin suppressed eosinophilic inflammation, suggesting their potential treating effect on allergic disorders [65].

Treatment with Qt (0.1–25 *μ*M, orally) blocked the airway epithelial cell IL-8 and monocyte chemoattractant protein- (MCP-) 1 expression by attenuating the signaling through a PI-3 kinase/protein kinase B (Akt)/nuclear factor (NF)-*κ*B pathway and inhibited chemokine expression. Also, Qt inhibited allergen sensitization, iMCP-1 expression, and airways hyperresponsiveness [136].

The inhibitory effects of Qt on different isotypes of immunoglobulins such as IgM, IgG, and IgA *in vitro* in mitogen-stimulated cells were also reported [137]. The effect of Qt and kaempferol on eicosanoid and nitric oxide-generating enzymes and its effect on the expression of proinflammatory genes were shown. Flavonoids in onions, such as Qt and kaempferol, also showed various antiviral, antimicrobial, anti-inflammatory, anticancer, and immunomodulatory activities [129, 138].

Kaempferol, the other compounds of onions (1–20 *μ*mol/L), inhibited eosinophil adhesion in activated airway epithelium at dose more than 10 *μ*mol/L in the TNF*α*-induced airway epithelium insult of six-week-old male BALB/c mice. Also, kaempferol reduced allergic and inflammatory airway diseases by NF-*κ*B signaling pathway [139]. In addition, kaempferol treatments attenuated the Th2-driven allergic airway disease in an experimental model of asthma induced by OVA challenge by decreasing the production of IL-5 and IL-13 and ameliorating airway hyperresponsiveness induced by OVA challenge. Kaempferol also inhibited IgE-mediated release of proinflammatory mediators from human mast cells [140].

Kaempferol suppressed OVA challenge-elicited airway inflammation by its immunomodulatory properties through antagonizing NF-*κ*B activation [86].

The inhibitory effect of kaempferol on LPS-induced epithelial eotaxin-1 expression and TNF-*α*-induced eosinophil-epithelial interaction was shown. Kaempferol also decreased eosinophil recruitment and accumulation in OVA-exposed mice.

Another flavonoid from onion, fisetin, inhibited IgE-mediated release of proinflammatory mediator and Th2-type cytokines from human mast cells and basophils [130]. S-allyl cysteine (SAC), a constituent of *A. cepa* (ranging from 10 to 600 *µ*mol/L), inhibited TNF-*α*-induced inflammation in splenocytes from asthmatic mice through inhibition of p38 and c-Jun N-terminal kinase (JNK) pathways and activation of extracellular signal-regulated kinase (ERK) [141].

#### 9.2.2. Clinical Evidence

Treatment of patients aged 18–85 years with allergic rhinitis and upper respiratory tract infection (URTI) with Qt (500 and 1000 mg/day) decreased nasal mucosal congestion, nasal itching, runny nose, sneezing attacks, and mucosal pallor [6]. In a randomized, double-blind clinical trial with 58 patients, treatment with Qt capsules (five capsules twice a day for 12 weeks) relieved perennial symptoms of allergic rhinitis. However, Qt treatment did not reduce serum IgE, and therefore, the mode of action of Qt in reducing symptoms of allergic rhinitis could not be concluded in this study [16].

Consumption of 20 *μ*M Qt and 20 *μ*M kaempferol in allergic rhinitis patients decreased the release of IL-8 and MIP-3*α* and reduced nasal mucosal congestion, nasal itching, runny nose, sneezing attacks, turbinate hypertrophy, and mucosal pallor [12]. Kaempferol supplementation (72 mg/kg) in inhaled maintenance therapy reduced TNF-*α* and IL-6, the inflammatory biomarkers in male smokers [113].

In a nutritional-based clinical trial on healthy adults, cruciferous vegetable diets including kaempferol 270 mg/kg, broccoli 30–72 mg/kg, and radish 38 mg/kg were administered to the individuals for 14 days. The results showed reduction of IL-6 and IL-8 indicating the immuno-regulatory effects of these compounds [56, 86, 109, 142].

Occupational asthma induced by garlic dust was evaluated in 12 subjects employed in the garlic growing and processing industry. Five of the seven patients indicated garlic-specific IgE levels more than 0.7 kU/L as well as increased onion-specific IgE levels.

**Figure 3:**
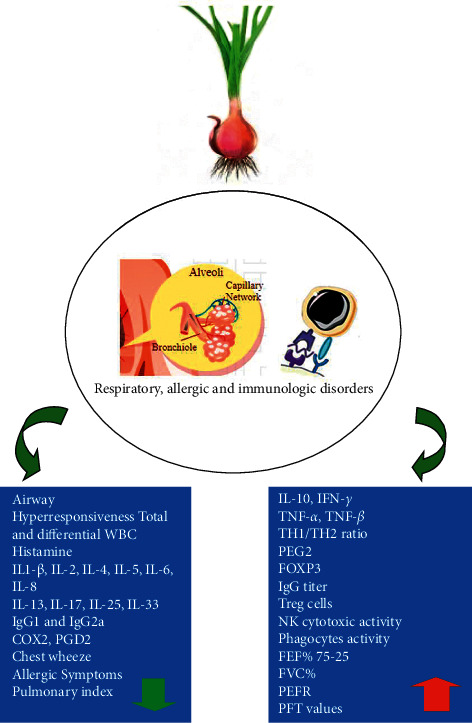
The possible molecular mechanisms of the preventive effects of *A. cepa* and its constituents on respiratory, allergic, and immunologic disorders.

**Figure 4:**
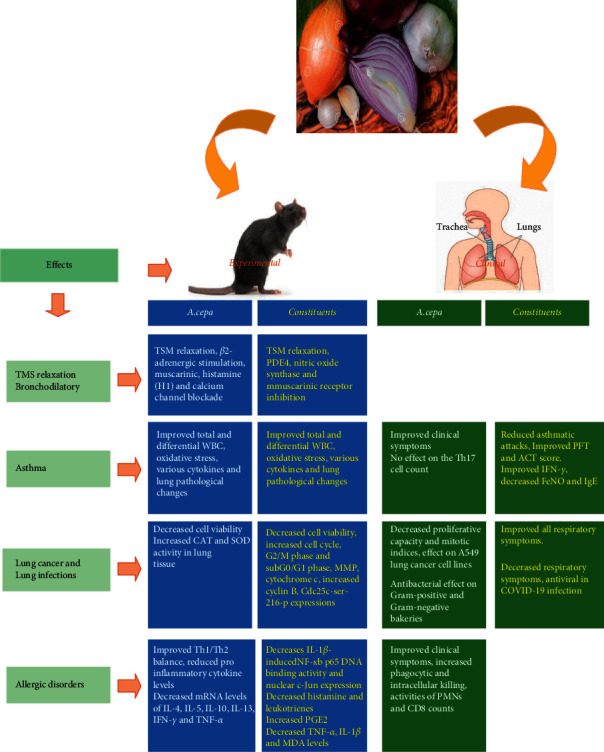
Experimental and clinical effects of *A. cepa* and its constituents on respiratory and allergic disorders.

Clinical studies also showed reduction in laboratory markers for allergy including TNF-*α* and IL-6 as well as IgE and eosinophil count in nasal discharge and allergic symptoms including nasal mucosal congestion, nasal itching, runny nose, sneezing attacks, turbinate hypertrophy, and mucosal pallor in allergic rhinitis induced by onion, Qt, and kaempferol. Treatment of allergic rhinitis patients with kaempferol also reduced TNF-*α*, IL-6, IL-8, IL-1ß, and MIP-3*α*. The effects of *A. cepa* and its constituents on allergic disorders are summarized in Table 5, and mechanisms involved in such effects are presented in Figure 3. Experimental and clinical effects of *A. cepa* and its constituents on respiratory and allergic disorders are also shown in Figure 4.

## 10. Discussion

In this article, the potential effects of *A. cepa* and its constituents on various respiratory disorders based on experimental and clinical findings were reviewed.

Various experimental studies showed the relaxant effects of *A. cepa* and its constituents mainly Qt on TSM. The relaxant effects of the plant and Qt were possibly mediated by different mechanisms including *β*2-adrenoceptor stimulation, muscarinic and histamine H_1_ receptor inhibition, calcium channel blocking, and phosphodiesterase enzyme-like mechanisms. These results suggest the possible bronchodilatory effects of the plant and Qt on obstructive respiratory diseases. However, further clinical studies are needed to examine this effect on asthma, chronic obstructive pulmonary diseases, or other obstructive pulmonary disorders.

Regarding the preventive effect of onion and its constituents on asthma, the AcE and the constituents of the plant mainly Qt decreased total and differential WBC in the blood and the BALF of animal models of asthma. Oxidant markers such as MDA were reduced, but antioxidants including CAT and SOD were increased in asthmatic animals by AcE and Qt. Serum and the BALF levels of PLA2, NF-*κ*B, PGD_2_, and GM-CSF were also decreased by AcE and Qt in the animal models of asthma. Treatment with the plant and Qt also decreased tracheal responsiveness and lung pathological changes in the sensitized animals. The level of IL-4 was decreased, but IFN-*γ* was increased and Th1/Th2 balance was improved in the animal models of asthma treated with AcE and Qt. Treatment with Qt also ameliorated asthma symptoms and protected asthma incidence in epidemiological studies. These results showed that *A*. *cepa* and its constituents could be considered possible preventive drugs for the treatment of asthma.

Treatment with the plant and Qt affects different cancer cell lines through modulating cell viability and other molecular mechanisms indicating their therapeutic effect on lung cancer. Clinical studies also support the effect of Qt on lung cancer.

The effect of extracts, essential oil, and the constituents of the plant, mainly Qt, on viral, microbial, parasitic, and fungal infections of the lung was shown. In experimental studies, beneficial effects of onion on lung infections caused by various viruses, bacteria, parasites, and fungi were reported. Treatment with Qt affects influenza virus infection and its lung manifestation. Clinical studies also showed the therapeutic effects of onion on symptoms of virus-infected flu. The effect of onion on the lung infected with *P. aeruginosa*, *S. aureus*, and *S. pneumonia* was also demonstrated. Treatment with Qt showed antiviral effects, and the effect of Qt on the treatment of COVID-19 patients was also indicated. The effect of Qt treatment on pulmonary tuberculosis was also demonstrated. Therefore, *A*. *cepa* and its constituents could be candidate drugs for treatment of various respiratory infections, especially viral infections and their lung manifestation mainly COVID-19.

Regarding the effect of onion and its constituents on allergic disorders, AcE treatment improved OVA-specific IgE, IL-4, IL-5, IL-10, IL-13, and IFN-*γ* levels in nasal mucosa and allergic symptoms in mouse models of allergic rhinitis; onion and its constituents inhibited cell proliferation, suppressed IL-2 and INF-*γ* gene expression in stimulated lymphocytes, and inhibited IgE-induced histamine and beta-hexosaminidase release from RBL-2H3 cells and production of IL-6, TNF-*α*, and IL-1*β* in murine macrophage cell lines. The plant also decreased VCAM-1 in fructose-fed rats and IL-6, IL-8, and TNF-*α* in APH condition. The plant and its constituents mainly Qt and kaempferol also decreased total and differential WBC and IL-4 in the blood and the BALF but increased IFN-*γ*, indicating enhanced Th1/Th2 balance both in the blood and the BALF of animal or cellular models of allergic disorders. The levels of IgM, IgG, and IgA in mitogen-stimulated cells and RANTES, MIP-1*β*, ECP, and MBP in the supernatants of cultured eosinophils from *M. corti*-infected mice were inhibited by Qt, and IL-5, IL-13, and IgE-mediated release of proinflammatory mediators was decreased by kaempferol. S-Allyl cysteine (SAC) also inhibited different cytokine gene expression in splenocytes of asthmatic mice and TNF-*α*-induced inflammation in HaCaT cells. Reduction in laboratory markers of allergy including TNF-*α* and IL-6, IL-8, IL-1ß MIP-3*α*, IgE, and eosinophil counts in nasal discharge and allergic symptoms including nasal mucosal congestion, nasal itching, runny nose, sneezing attacks, turbinate hypertrophy, and mucosal pallor in allergic rhinitis was decreased by the plant, Qt, and kaempferol in clinical studies. However, induction of allergic reaction to onion was indicated in a number of studies.

The current review article therefore indicates possible bronchodilatory and preventive effects of onion and Qt on asthma and other obstructive respiratory diseases. The effects of the plant and its constituents on lung cancer, lung infections, and allergic disorders were also reported both in experimental and clinical studies. However, before preparing drugs based on *A. cepa* and its constituents for clinical practice, further standard clinical trials are needed to be performed.

## Figures and Tables

**Table 1:** Different constituents of *A. cepa*.

Major constituents	Other constituents	References
Water		[51]
Proteins		[51, 94]
Carbohydrates	Inulin, fructooligosaccharides, isorhamnetin-4-glucoside, galactose, glucose, and mannose	[51]
Vegetal hormone lectin	Glycoquinine	[13]
Steroids	Catechol, protocatechuic acid, thiocyanate, and thiopropiono aldehyde	[51, 94, 143]
Phytoestrogens	Coumestrol, zearalenol, isoflavones, and humulone	[51, 94, 143]
Vitamins	A, B complex, C, and E	[51, 94, 143]
Minerals	Selenium, phosphorus, iron, calcium, and chromium	[51, 94, 143]
Flavonoids	Quercetin, apigenin, rutin, myricetin, kaempferol, catechin, resveratrol, epigallocatechol-3-gallate, luteolin and genistein, quercetin aglycone, quercetin diglucoside, quercetin 4-glucoside, and isorhamnetin monoglycoside or kaempferol monoglycoside	[17, 51, 94, 144]
Organosulfuric compounds	Thiosulphinates, cepaenes, cysteine, S-methyl cysteine sulfoxide, diallyl disulfide, allyl methyl sulfide, allyl propyl disulfide, gamma-L-glutamyl-trans-S-1-propenyl-L-cysteine sulfoxide, S-propenyl cysteine sulfoxide, S-alk(en)yl cysteine sulfoxides, and S-allyl cysteine sulfoxide	[51, 145]
Allicin	Diallyl disulfide, diallyl trisulfide, and ajoene	[13, 51, 143, 146]
Phenolic compounds	Phenolics, phenolic acids, anthocyanins, and hydroxycinnamic acid	[42, 51]
Lipophilic antioxidants	Dialkyl disulfides, aglycones, anthocyanin, saponins, and fistulosin (octadecyl 3-hydroxyindole)	[41, 62]

**Table 2:** The possible bronchodilatory effects of *A. cepa* and its constituents on the tracheal smooth muscle and its possible mechanisms.

Study type	Study design	Preparations	Dose	Effects	Ref.
Exp	Murine TSM contracted with Cch	*A. cepa* extract	10, 100, and 1000 *μ*g/ml	Relaxing activity on TSM	[30]
	Rat TSM contracted with Mch and KCl	*A. cepa* extract	2, 4, 8, 16, 32, and 64 mg/ml	*β*2-Adrenergic stimulatory and calcium channel blockade mechanisms	[61]
	Rat trachea contracted with Cch	*A. cepa* peel	3–30 mg/kg	Inhibition of Ca^2+^ channels and phosphodiesterase enzyme-like mechanisms	[85]
	Animals 30 min prior to allergen inhalation	Ethanolic extracts	10 *µ*l/1 ml, orally	Relation effect on TSM	[86]
	A/J mice precontracted with Cch	Qt	3.5, 7.5, and 15 *μ*g/ml	Relaxing activity on TSM; inhibited Ca^2+^-permeant LVDCCs, TRPC3, and STIM/Orai channels	[87]
	TSM of mice	Qt	100 nM-1 mM and 50 *μ*M	Relaxant effect on TSM; PDE4 inhibition	[88]
	OVA-sensitized guinea pigs	Qt	20 mg/kg	Relaxant effect on TSM *in vivo* and *in vitro*	[89]
	Cch-induced TSM contractions	Qt	10^−6^−3 × 10^−4^ M	Inhibited nitric oxide synthase; *N*^G^-nitro-L-arginine methyl ester significantly reduced the effect of Qt	[147]

Ref.: references, Exp: experimental, Clin: clinical, TSM: tracheal smooth muscle, OVA: ovalbumin, TQ: thymoquinone, and Cch: carbachol.

**Table 3:** The preventive effects of *A. cepa* and its constituents on asthma.

Study type	Study design	Preparations	Dose	Effects	Ref.
Exp	Murine model of asthma	*A. cepa* extract	10, 100, or 1000 *μ*g/mL, orally	Decreased recruitment of eosinophils and their activation in the lungs	[30]
	OVA-sensitized rat	*A. cepa* aqueous extract	150 and 300 mg/kg b.w.	Decreased cellular infiltration and lung inflammation	[17]
	OVA-sensitized rat	*A. cepa* aqueous extract	150 and 300 mg/kg	Decreased eosinophil and lymphocyte counts in blood and the BALF; inflammation was reduced	[91]
	BT-sensitized A/J mice	Qt	3.5, 7.5, and 15 *μ*g/ml	Reduced total number of cells in the BALF; anti-inflammatory and immunomodulatory effects	[30]
	OVA-sensitized BALB/c mice	Isoquercitrin and Qt	15 mg/kg and 10 mg/kg	Decreased blood neutrophils, lung homogenate IL-5, and eosinophilic inflammation	[112]
	Airway epithelial cells	Qt	0.1–25 *μ*M, orally	Decreased airway epithelial cell, IL-8, and MCP-1 expression by attenuating signaling through a PI-3 kinase/Akt/NF-*κ*B pathway; inhibited chemokine expression	[136]
	OVA-sensitized guinea pigs	Qt	20 mg/kg	Decreased TR	[60]
Clin	Asthmatic patients	*A. cepa* extract	—	Improved clinical symptoms	[128]
	Asthmatic adult	*A. cepa* extract	15 mg/kg/day	Improved clinical symptoms; no effect on the Th17 cell count	[148]
	Asthmatic patients	Kaempferol	—	Reduced asthmatic attacks, improved FPT and ACT score	[126]
	Asthmatic patients	Thiosulfinates	500 mg	Improved FPT and ACT score; increased FEF 25–75% and FEV 1% and IFN-*γ*; decreased FeNO and IgE	[86, 142]
	Asthmatic patients	Kaempferol	0.90 ± 0.07 *μ*g/L	Reduced asthmatic attacks; increased FVC and FEV1%	[149]

Ref.: references, Exp: experimental, Clin: clinical, TSM: tracheal smooth muscle, OVA: ovalbumin, TQ: thymoquinone, Cch: carbachol, Qt: quercetin, BALF: bronchoalveolar fluid, BT: *Blomia tropicalis*, [MCP]-1: monocyte chemoattractant protein, TR: tracheal responsiveness, FEV: forced expiratory volume, IFN-*γ*: interferon gamma, and FVC: forced vital capacity.

**Table 4:** The preventive effects of *A. cepa* and its constituents on lung cancer and lung infections.

Study type	Study design	Preparations	Dose	Effects	Ref.
	Rats exposed to nicotine	*A. cepa* oil	100 mg/kg b.w., 21 days	Increased CAT and SOD activity in the lung tissue	[101]
Exp	Mice	Qt	—	Decreased superoxide radicals and LPO; lung morphological changes	[110]
	Swiss albino male mice	Qt	—	Lowered the lipid peroxide levels	[111]
	Cell line NCI-H209	Qt glucuronides	—	Qt glucuronides inhibited proliferation through G2/M arrest of the cell cycle and induced apoptosis via caspase-3 cascade in the human lung cancer cell line NCI-H209	[99]
	Cat fish	*A. cepa* extract	1000 mg/ml	Antibacterial activity	[98]
	Mice	Ethanol extract	10 and 20 mg/ml	Antibacterial effect	[150]
	*Mesocestoides corti* infection in BALB/c mice	Qt	5.0–20.0 mg/kg, oral	Decreased peripheral blood eosinophilia and IgE hyperproduction	[135]
Clin	Lung cancer patients	*A. cepa* extracts	10 g L^−1^	Antiproliferative capacity; decreased mitotic indices	[98]
	Adenocarcinoma patients	Qt-rich food	—	Decreased lung cancer risk; upregulated GSTM1, GSTM2, GSTT2, and GSTP1; downregulated specific P450 genes	[102]
	Lung cancer patients	Qt-rich food	mg/100 g	Decreased carcinogenesis-related miR-146, miR-26, and miR-17 families	[151]
	DPT patients	Qt and QP	5 g in 100 mL saline	Reduction of the disease manifestation	[115]
	Cell line NCI-H209	Qt glucuronides	—	Qt glucuronides inhibited proliferation through G2/M arrest of the cell cycle and induced apoptosis via caspase-3 cascade in the human lung cancer cell line NCI-H209	[99]
	Clinical study	*A. cepa* aqueous extract	—	Antibacterial effect on Gram-positive and Gram-negative bacteria; effect on A549 lung cancer cell lines	[100]
	Virus-infected patients	Inhaled onion, garlic extract	—	Improved all respiratory symptoms	[107]
	Clinical study	Vitamin C and Qt	30 or 40 mg/kg, 4 days	Treatment of respiratory tract infections with COVID-19	[112]
	Clinical study	Qt and NAC, nebulized	600 and 300 mg/mL for Qt and NAC	Decreased respiratory symptoms; antiviral in COVID-19 infection	[114]

Ref.: references, Exp: experimental, and Clin: clinical.

**Table 5:** The effects of *A. cepa* and its constituents on allergic and immunologic disorders.

Study type	Plant preparations	Dose	Study models	Effects	Ref.
Exp	*A. cepa* extract	100 and 1,000 mg/kg/day	Antiallergic immune response	Improved Th1/Th2 balance; reduced proinflammatory cytokine levels	[131]
	*A. cepa* extract	150 and 300 mg/kg b.w,	Intranasal	Decreased cellular infiltration and eosinophil and lymphocyte count in the blood and BALF	[91]
	*A. cepa* extract	Onion 20 and 40 *μ*l	Intranasal	Decreased mRNA levels of IL-4, IL-5, IL-10, IL-13, IFN-*γ*, and TNF-*α*	[119]
	*A. cepa* extract	10 mg/400 mL	Peritoneal cells	Decreased cytokine release, macrophage phagocytic activity, and NK cell activity	[152]
	*A. cepa* extract	20 *μ*L	Lung eosinophilia infiltration	Reduced inflammatory cytokines and total cell counts in BALF and EPO in the lungs	[31]
	*A. cepa* extract	500 mg/kg	Lung and tissue	Decreased no, IL-6, TNF-*α*, IL-1*β* levels	[153]
	*A. cepa* extract	500 and 50 mg/kg/day	Pulmonary tissues	Low doses of onion was not toxic but high dose was toxic to rats	[154]
	*A. cepa* extract	250–600 mg	Allergic rhinitis	Reduced production of IL-4, IL-5, and IL-13	[30]
	Kaempferol	75 mg/kg	OVA-sensitized mice	Reduced Th2, Th17, IL-4, IL-17, and TGF-*β* but increased Treg cells, Th1/Th2 ratio, FOXP3, IFN-*γ*/IL-4 ratio, and IFN-*γ* gene expressions	[155]
	Kaempferol	20 *μ*mol/l	OVA-sensitized mice	Decreased IL-1*β*-induced NF-*κ*b p65 DNA-binding activity and nuclear c-Jun expression	[156]
	Kaempferol	—	OVA-sensitized rats	Reduced eosinophil count, IgE, IL-4, IL-5, IL-13, TNF-*α*, and IFN-*γ* levels	[157]
	Kaempferol	50 *µ*M	OVA-sensitized rats	Inhibited LTB4 production without cytotoxicity	[158]
	SAC	10 or 20 mg/kg	OVA-sensitized mice	Reduced airway hyperresponsiveness, inflammatory cell counts, Th2-type cytokines in BALF, and serum OVA-specific IgE	[159]
	SAC	—	C57BL/6 mice	Alleviated clinical symptoms; improved TNF-*α*, IL-17, ADNP, MAP1LC3A, and MMP-9 levels	[160]
	SAC	—	MISD in mice	Inhibited TNF-*α*-induced activation of p38, JNK, and NF-*κβ* pathways	[161]
	Kaempferol	0.46 g	OVA-sensitized guinea pigs	Decreased histamine and leukotrienes; increased PGE2	[162]
	Amentoflavone	—	Mouse ear and rat paw edema	Inhibited phospholipase A2 and cyclooxygenase pathway	[163]
	Quercetin	30 mg/kg	Bone marrow-derived mast cells	Decrease allergen-induced airway hyper responsiveness, Th2 responses in the lung, lung eosinophilia, and goblet cell metaplasia	[164]
	Kaempferol	10 or 20 *μ*M	Knee osteoarthritic rats	Decreased TNF-*α*, IL-1*β*, and MDA levels	[165]
	Kaempferol	25, 50, 100, and 200 *μ*m	Rat articular chondrocytes cultures	Reduced interleukin-1*β*-stimulated proinflammatory mediators by inhibiting the NF-*κβ* pathway	[166]
	FFR + onion	150 and 400 mg/kg/d	Wistar rats	Decreased heart ENOS and VCAM-1	[125]
	Kaempferol	2 mg/kg/day or 10 mg/kg/day	Rat gingival tissues	Reduced INOS and TNF-*α* expression and nuclear p6; increased cytosolic p65; downregulated Erk, p38, JNK, and NIK/IKK expression	[167]
	Quercetin and kaempferol	10 and 20 *μ*M	Rbl-2h3 intestinal cells	Inhibited the secretion of TNF-*α* and IL-4 in antigen-stimulated RBL-2H3 cells	[41]
	SAC	10 to 600 *µ*mol/L	Splenocytes of asthmatic mice	Inhibited TNF-*α*-induced inflammation in HaCaT cells	[141]
	Thiosulfonates	100 mg/kg	Guinea pig model of asthma	Inhibited inflammatory cell influx by thiosulfinates	[95]
	Kaempferol	1–20 *μ*mol/l	Asthmatic mice	Inhibited eosinophil adhesion to TNF-*α*-activated airway epithelium	[140]
	Kaempferol	0.22 *μ*m	BALB/c mice	Inhibited IL-4-induced transcription factor STAT6 activation by specifically targeting Janus kinase 3	[139]
	*Polygonum tinctorium* Lour.	0.2, 2, or 20 mg/kg	OVA-induced mouse asthma	Reduced caspase-1 activity in nasal mucosa, IL-32, and IL-8	[168]
	Kaempferol	20 *μ*m	BALB/c mice model of asthma	Suppressed LPS-induced IL-8 through the TLR4 activation; inhibited eotaxin-1 induction	[169]
	Kaempferol	—	LPS-stimulated THP-1 cells	Suppressed LPS-induced MDC, IP-10, IL-8, Th1, Th2, and neutrophil-related chemokines	[170]
	Kaempferol		Mouse model of asthma	Deceased inflammatory cell invasion, goblet cell hyperplasia, mucus secretion, regulated TNF-*α*, IL-4, IL-5, and IL-13	[171]
	Quercetin	40 *μ*M	Allergic asthma	Inhibited rat tracheal contractility	[59]
	Quercetin	8 or 16 mg/kg	Allergic asthma BALB/c	Decreased allergic airway inflammation and hyperresponsiveness; improved Th1/Th2 balance via the suppression of GATA-3 and increase of T-bet expression	[32]
	Quercetin	25 mg/kg	Allergic asthma BALB/c	Inhibited nasal rubbing movements and sneezing	[172]
	Quercetin	20 mg/kg	Allergic asthma BALB/c	Inhibited nasal symptoms; increased TRX levels in nasal lavage fluids	[173]
Clin	*A. cepa* extract	Onion juice 52 g	Neuronal cell oxidative stress	Improved clinical symptoms; increased phagocytic and intracellular killing activities of PMNs and CD8 counts	[81]
	*A. cepa* extract	—	Allergic rhinitis	Reduced IgE and eosinophil count in nasal discharge; stabilized mast cell membranes	[127]
	*A. cepa* extract	—	Allergic disorders	Relieving perennial allergic rhinitis symptoms; increased phagocytic and intracellular killing activities of PMNs and CD8 counts	[174]
	*A. cepa* extract	50 g	Allergic induction	Severe allergic reactions induced by cooked onion	[175]
	*A. cepa* extract	—	Allergic hypersensitivity	Improved clinical symptoms	[128]

Ref.: references, Exp: experimental, Clin: clinical, IFN-*γ*: interferon- *γ*, TNF-*α*: tumor necrosis factor-*α*, BALF: bronchoalveolar lavage fluid, EPO: eosinophil peroxidase, BAL: bronchoalveolar lavage, MDA: malondialdehyde, NO_2_: nitrite, NO_3_: nitrate, SOD: superoxide dismutase, CAT: catalase, TC: total cholesterol, LDL-C: low-density lipoproteins, TG: triglycerides, HDL-C: high-density lipoproteins, PPAR *γ*: peroxisome proliferator-activated receptor *γ*, TSM: tracheal smooth muscle, i.p.: intraperitoneally, MMP-9: matrix metalloproteinase 9, MAP1LC3A: microtubule-associated proteins 1 A/1 B light chain 3A, ADNP: activity-dependent neuroprotector homeobox, ENOS: endothelial nitric oxide synthase, VCAM-1: vascular cell adhesion molecule-1, IL-17: interleukin 17, PGE2: prostaglandin E2, LTB4: leukotriene B4, THP-1: human monocytic cell line, OVA: ovalbumin, C57BL: C57 black 6, LPS: lipopolysaccharide, SAC: S-allyl cysteine, and MISD: multiple inflammatory skin diseases.
